# Prosthetic maintenance profiles and functional performance of single-implant mandibular overdentures in geriatric edentulism: A prospective clinical evaluation

**DOI:** 10.6026/973206300222466

**Published:** 2026-04-30

**Authors:** Deepinder Kaur, Rishmreet Kaur, Mamta Garg, Samir Mansuri, Rushit Patel, Rajesh K.V, Rahul Tiwari, Prashant M.C

**Affiliations:** 1 Department of Dental surgery, Guru Nanak Dev Dental College and Research Institute, Sunam, Punjab, India; 2 Consultant Oral and Maxillofacial Surgeon, Ahmedabad, Gujarat, India; 3 Department of Oral & Maxillofacial Surgery, Karnavati School of Dentistry, Uvarsad, Gandhinagar, Gujarat, India; 4 Department of Prosthodontics, Anil Neerukonda Institute of Dental Sciences, Visakhapatnam andhra Pradesh, India; 5 Department of Oral and Maxillofacial Surgery, RKDF Dental College and Research Centre, Sarvepalli Radhakrishnan University, Bhopal, Madhya Pradesh, India

**Keywords:** Dental implants, overdentures, aged, prosthesis retention, treatment outcome

## Abstract

Two-implant overdentures have long been regarded as the gold standard for mandibular edentulism. Hence, prosthesis maintenance, and patient satisfaction-all of which were evaluated over a two-year follow-up-requires prospective evaluation. We found that locator attachment wear was the most frequent prosthetic problem (17.5%) in our study. A smaller fraction had denture fractures and relining requirements, while 15% of patients needed changes because of retention loss. Thus, prosthetic profile can be achieved with regular maintenance.

## Background:

Edentulism is highly prevalent among geriatric individuals and is frequently associated with diminished masticatory efficiency, nutritional compromise and reduced oral-health-related quality of life [1]. Conventional mandibular dentures often perform poorly in elderly patients due to progressive ridge resorption and diminished neuromuscular coordination, resulting in instability, discomfort and repeated prosthetic adjustments [2]. Implant-retained overdentures were introduced to overcome these limitations and several clinical studies have reported superior functional outcomes and improved oral comfort when compared with traditional complete dentures [3, 4]. While the two-implant overdenture remains widely accepted as a standard of care, the financial, anatomical and surgical constraints common in geriatric populations have generated interest in simplified alternatives, including single-implant-supported overdentures [5]. Evidence suggests that a single midline implant can provide adequate anchorage with reduced morbidity and may minimize long-term biological and technical complications [6, 7]. However, clinical success in this age group also depends on prosthetic maintenance requirements, attachment wear and functional performance, factors that are increasingly recognized as critical to long-term patient satisfaction and treatment sustainability [8]. Therefore, it is of interest to report the prosthetic maintenance profile and functional performance of single-implant mandibular overdentures in geriatric edentulous patients, offering clinically relevant insights for practitioners managing elderly rehabilitation.

## Materials and Methods:

This prospective clinical study included a total sample of 40 geriatric patients aged 65 years and above who were completely edentulous in the mandible and reported persistent difficulties with conventional dentures. Patients were selected based on adequate bone availability in the mandibular symphysis and medical stability and all provided informed consent prior to participation. A single endosseous implant was placed in the midline region following a standardized surgical protocol under local anesthesia and osseointegration was allowed before incorporating a locator attachment system into the existing mandibular denture. At each scheduled follow-up visit, prosthetic maintenance requirements were documented, including attachment wear, relining needs, denture base adjustments and any mechanical complications. Functional performance was evaluated using patient-reported outcome measures assessing retention, comfort, mastication and overall usability of the prosthesis. Retention force was measured using a digital gauge and all clinical findings were recorded systematically. Data were analyzed using descriptive and appropriate inferential statistics, with significance set at p < 0.05.

## Results:

Retention forces increased steadily from baseline (11.2 N) to 12 months (13.1 N), with statistical significance. The values stabilized thereafter, indicating durable retention over the two-year period. The consistent improvement suggests that single-implant overdentures provide reliable functional stability, reducing denture displacement during mastication and speech. This retention gain is clinically relevant for elderly patients, as it directly enhances chewing efficiency and confidence in social situations, thereby contributing to overall prosthetic success. Prosthetic complications were relatively infrequent, with locator attachment wear being the most common (17.5%). Denture fractures and relining needs were observed in a smaller subset, while 15% of patients required adjustments due to loss of retention. These findings suggest that while minor technical issues are expected, they are manageable through routine maintenance. Importantly, no major prosthetic failures occurred, supporting the practicality of this simplified implant approach in geriatric populations with limited tolerance for complex interventions Table 1 (see PDF). Patient-reported outcomes showed significant improvement across all domains. Retention and overall satisfaction demonstrated the greatest gains, rising from low baseline scores (~3.5) to high post-treatment ratings (~8.7). Mastication and comfort also improved markedly, reflecting enhanced functional efficiency and reduced discomfort. Speech clarity showed moderate yet sustained improvement. These results highlight the impact of a single-implant overdenture on daily life, underscoring its ability to restore confidence, improve diet variety and elevate psychosocial well-being in elderly individuals Table 2 (see PDF).

## Discussion:

The present prospective clinical study demonstrated that single-implant mandibular overdentures provide substantial improvements in prosthetic retention, functional performance and patient satisfaction in geriatric edentulous individuals. Progressive improvement in retention forces and consistent enhancement in patient-reported outcome measures suggest that the placement of a single midline implant can significantly improve the stability and usability of mandibular dentures. These results align with the growing body of literature indicating that simplified implant-supported prosthetic approaches are particularly advantageous for elderly populations who may have systemic limitations, anatomical constraints, or financial barriers to more extensive implant rehabilitation [9, 10]. Retention is widely regarded as one of the most critical determinants of overdenture success. In the present study, retention forces increased significantly during the first year following implant loading and remained stable throughout the follow-up period. This pattern suggests effective osseointegration and consistent mechanical performance of the locator attachment system. Similar findings have been reported in previous clinical investigations evaluating implant-retained overdentures, which have demonstrated that midline symphyseal implants can effectively withstand functional masticatory loads and significantly enhance denture stability [9]. De Resende *et al.* also reported that locator attachments provide durable retention and predictable clinical outcomes, making them a preferred option for overdenture therapy in elderly patients [10]. Prosthetic maintenance represents another important parameter in evaluating implant overdenture success. In the present study, prosthetic complications were relatively infrequent, with locator attachment wear being the most common maintenance event. Occasional relining requirements and minor denture fractures were also observed but were easily managed through routine follow-up care. These observations correspond with previous studies demonstrating that implant-retained overdentures generally exhibit low rates of technical complications when appropriate attachment systems and maintenance protocols are employed [11, 12]. Velasco-Ortega *et al.* similarly reported that implant overdentures provide reliable functional performance with manageable prosthetic maintenance requirements over extended follow-up periods [12]. Patient satisfaction and oral-health-related quality of life improved markedly following treatment in the present cohort. Improvements were particularly evident in retention, mastication and overall comfort scores, reflecting enhanced prosthetic stability and functional efficiency. These findings are consistent with previous reports indicating that improved denture stability has a significant positive impact on the psychosocial well-being and nutritional status of elderly individuals [13]. Studies investigating implant-retained overdentures have consistently demonstrated significant improvements in chewing ability, speech and patient confidence compared with conventional complete dentures [14, 15]. Recent studies focusing specifically on single- implant mandibular overdentures further support the findings of the present investigation. Chauhan *et al.* conducted a prospective clinical study evaluating geriatric patients rehabilitated with single-implant overdentures and reported significant improvements in retention and patient satisfaction after treatment, with high implant survival rates and acceptable complication profiles [16]. Similarly, a systematic review by Tudts *et al.* comparing overdentures retained by one versus two implants concluded that single-implant overdentures can achieve favourable functional outcomes and represent a viable alternative when patient-related constraints limit the use of multiple implants [17]. Long- term evidence also supports the clinical reliability of this treatment modality. Yazigi *et al.* reported a fifteen-year follow-up study of single midline implant-supported overdentures and demonstrated excellent implant survival and sustained prosthetic function over extended observation periods [18]. Their findings suggest that simplified implant protocols can maintain predictable outcomes even over long durations when appropriate prosthetic design and maintenance strategies are implemented. In addition to retention and functional improvements, patient-centered outcomes such as oral comfort and quality of life have become increasingly important measures of treatment success. Ibrahim *et al.* reported significant improvements in patient satisfaction and masticatory efficiency among individuals treated with implant-supported overdentures, highlighting the clinical importance of prosthesis stability in restoring oral function [19]. Furthermore, Elezaby *et al.* demonstrated that implant-retained overdenture therapy can influence peri-implant bone stability and occlusal loading patterns, emphasizing the importance of careful prosthetic planning in ensuring long-term implant success [20]. Overall, the findings of the present study support the growing consensus that single-implant mandibular overdentures represent a predictable, minimally invasive and cost-effective treatment option for geriatric edentulous patients. By improving denture retention, functional performance and patient satisfaction while maintaining relatively low complication rates, this treatment approach offers a practical solution for elderly individuals who may not be suitable candidates for more extensive implant rehabilitation. Nevertheless, several limitations should be acknowledged. The relatively modest sample size and limited follow-up duration may restrict the generalizability of the findings. Long-term multicenter studies with larger patient populations are required to further evaluate the biological and prosthetic outcomes associated with single-implant overdenture therapy over extended periods.

## Conclusion:

Single-implant mandibular overdentures demonstrated favourable functional performance and required only modest, predictable prosthetic maintenance in geriatric edentulous patients. The improvements in comfort and usability, combined with manageable attachment-related interventions, support the clinical practicality of this simplified treatment approach. These data show single-implant overdentures as a viable, cost-effective option for elderly individuals who may not be suitable for more extensive implant rehabilitation.
